# Morbidity, mortality and missed appointments in healthcare: a national retrospective data linkage study

**DOI:** 10.1186/s12916-018-1234-0

**Published:** 2019-01-11

**Authors:** Ross McQueenie, David A. Ellis, Alex McConnachie, Philip Wilson, Andrea E. Williamson

**Affiliations:** 1 General Practice and Primary Care, School of Medicine, Dentistry and Nursing, MVLS, University of Glasgow, Glasgow, G12 9LX UK; 2 Department of Psychology, Lancaster University, Lancaster, UK; 3 Robertson Centre for Biostatistics, Institute of Health and Wellbeing, MVLS, University of Glasgow, Glasgow, UK; 4 Centre for Rural Health, Institute of Applied Health Sciences, University of Aberdeen, Aberdeen, UK

**Keywords:** Missed appointments, primary care, health utilisation, health promotion, health inequalities, social vulnerability, administrative data, long-term conditions, morbidity, mortality

## Abstract

**Background:**

Recently, studies have examined the underlying patient and practice factors for missed appointments, but less is known about the impact on patient health. People with one or more long-term conditions who fail to attend appointments may be at risk of premature death. This is the first study to examine the effect of missed primary healthcare appointments on all-cause mortality in those with long-term mental and physical health conditions.

**Methods:**

We used a large, nationwide retrospective cohort (*n* = 824,374) extracted from routinely collected general practice data across Scotland over a 3-year period from September 2013 until September 2016. This data encompasses appointment history for approximately 15% of the Scottish population, and was linked to Scottish deaths records for patients who had died within a 16-month follow-up period. We generated appointment attendance history, number of long-term conditions and prescriptions data for patients. These factors were used in negative binomial and Cox’s proportional hazards modelling to examine the risk of missing appointments and all-cause mortality.

**Results:**

Patients with a greater number of long-term conditions had an increased risk of missing general practice appointments despite controlling for number of appointments made, particularly among patients with mental health conditions. These patients were at significantly greater risk of all-cause mortality, and showed a dose-based response with increasing number of missed appointments. Patients with long-term mental health conditions who missed more than two appointments per year had a greater than 8-fold increase in risk of all-cause mortality compared with those who missed no appointments. These patients died prematurely, commonly from non-natural external factors such as suicide.

**Conclusions:**

Missed appointments represent a significant risk marker for all-cause mortality, particularly in patients with mental health conditions. For these patients, existing primary healthcare appointment systems are ineffective. Future interventions should be developed with a particular focus on increasing attendance by these patients.

**Electronic supplementary material:**

The online version of this article (10.1186/s12916-018-1234-0) contains supplementary material, which is available to authorized users.

## Background

Healthcare systems should manage and treat chronic, long-term conditions (LTCs) effectively. However, while traditional models of care focus on the treatment of individual conditions, multimorbidity – the presence of two or more LTCs [1] – is increasing in prevalence [2, 3]. Greater numbers of LTCs are associated with deprivation [4], older age [5], high mortality [6] and depression [7]. High levels of multimorbidity are also correlated with increased treatment burden [8], and repeated attendance at primary healthcare appointments is required to avoid adverse outcomes in conditions such as diabetes [9]. We recently described an association between demographic risk factors and a pattern of repeated missed primary care appointments [10], which raises the question of whether repeatedly missed appointments are associated with greater numbers of LTCs, potentially further increasing the overall burden of disease as well as socioeconomic health inequalities. Missed appointments may thus represent one aspect of unmet health needs, yet there has been no prior published research in this area.

Herein, we used a large Scottish primary healthcare appointment dataset to quantify the association between number of LTCs contributing to multimorbidity – both physical and mental health related – and the risk of missing general practice appointments. We also used data linkage with the Scottish death registry to quantify the risk of all-cause mortality and describe the causes of death in patients who miss multiple appointments, allowing, for the first time, a quantitative examination of the association of missed appointment patterns (as a proxy for unmet medical need) with adverse outcomes.

## Methods

### Study design

National Health Service (NHS) general practices provide healthcare for most of the UK population. Patients are registered at a single practice, and are able to schedule appointments at their own discretion. Because practices generally control access into treatment services in the UK health system and hold data on almost all health service encounters, they allow the examination of the association between appointment attendance patterns and LTCs.

Routinely collected NHS data from general practices across Scotland between September 5, 2013, and September 5, 2016, were extracted with agreement from participating practices. Recruitment methods and the characteristics of the practices are described in a previous protocol paper [11]. Overall, 136 practices from 11 Scottish health boards took part, resulting in a cohort of 11,490,537 separate appointments from 824,374 patients.

Letters of comfort were issued by the West of Scotland NHS Ethics Committee and the University of Glasgow College of Medical, Veterinary & Life Sciences Ethics Committee, confirming that the full study did not need NHS ethics permission. Public Benefit and Privacy Panel approval was granted by NHS Information Services Scotland in December 2016. Data were aggregated where necessary to ensure individual patient privacy.

Due to the sensitive nature of NHS administrative data, the datasets generated or analysed during the present study will not be made publicly available. Data have been made available only to the research team under controlled access and strictly for the purposes of this research study. These data were stored in an NHS Safe Haven – a platform allowing researchers to analyse confidential patient data securely. Summary data, at the level of disclosure-checked output from the National Safe Haven, and coding can be obtained from the corresponding author on reasonable request. Categories of variables used in this paper are given in Additional file 1.

### Outcomes

Our primary analysis assessed whether patients with higher numbers of LTCs missed more general practice appointments. A secondary analysis considered whether patients who missed multiple appointments were at a higher risk of all-cause mortality.

### Data analysis

Patient and practice level data used in this study were prepared as described previously [10]. Briefly, patients were categorised into attendance categories averaged over the 3-year study period from September 5, 2013, until September 5, 2016, as follows: zero missed appointments (zero group); low number of missed appointments, < 1 per year (low group); medium number of missed appointments, 1–2 per year (medium group); and high number of missed appointments, > 2 per year (high group).

LTC data were derived from patients’ primary care Read codes and included both priority 1 and priority 3 codes [12]. LTC counts were calculated using the 43 LTCs as described by Barnett et al. [4], with refinement of codes included in addiction/mental health categories. These represent the LTCs with prevalence above 0.1% in the UK population. Prescription data were used when available to allocate LTCs to a patient if Read codes were not adequately recorded [4]. LTCs were further categorised as related to mental or physical health, using the approach adopted by Barnett et al. [4].

We used a negative binomial model to examine risk of missing appointments, controlling for both patient-based factors – age, sex, socioeconomic status and distance – and practice level factors, including average interval between booking and appointment (appointment delay), average appointment time per patient, average total number of appointments offered to each patient over 3 years, practice rurality and mean practice socioeconomic status. This model controlled for number of appointments made by including it as an offset.

Similarly, we used Cox’s proportional hazards controlling for these factors to examine all-cause mortality as an outcome. All-cause premature mortality was evaluated by linking patient community health index numbers – a unique identifier for each patient – from our initial dataset to Scottish death records databases using the follow-up period between September 6, 2016, and December 31, 2017. These identifiable community health index numbers were then anonymised by Albasoft, an NHS trusted third party used for data extraction on this project, before upload to the Safe Haven.

## Results

### Patient demographic factors

Using our retrospective cohort of general practice data across Scotland, we established a dataset containing 11,490,537 separate consultations (*n* = 824,374 patients) for analysis. Table 1 reports patient demographic factors for none, one to three, and four or more LTCs. Overall, 59.0% of participants had one or more LTCs, with 13.3% reporting four or more. However, only 7.0% of those in the ‘zero’ group had four or more LTCs, whilst in the ‘high’ group, 40.1% of participants had four or more. Furthermore, high levels of LTCs were associated with deprivation, wherein 64.3% of those in the most deprived quantile had one or more LTC, compared to 51.1% of those in the most affluent quantile.

**Table 1:** Patient demographic factors for categories of long-term conditions (LTCs). Missed appointment category was defined as average annual rate of missed appointments over a 3-year period, as follows: zero, 0; low, < 1; medium, 1–2; high, > 2

	No LTCs *n* = 338,372 (41.1%)	One to three LTC *n* = 375,893 (45.7%)	Four plus LTC *n* = 109,579 (13.3%)	Overall *n* = 824,374
Missed appointment category; missing values *n* = 0				
Zero	226,190 51.5%	182,682 41.6%	30,720 7%	439,592 100%
Low	84,556 37%	111,928 49%	31,881 14%	228,365 100%
Medium	22,157 22.8%	51,569 53.1%	23,351 24.1%	97,077 100%
High	5819 9.8%	29,714 50.1%	23,807 40.1%	59,340 100%
Age; missing values *n* = 6650 (0.8%)				
0–15	100,880 80.8%	25,910 19.2%	22 0%	134,732 100%
16–30	95,785 56.7%	70,320 41.7%	2686 1.6%	168,791 100%
31–45	70,040 41.1%	89,731 52.7%	10,622 6.2%	170,393 100%
46–60	41,401 24.1%	103,869 60.5%	26,330 15.3%	171,600 100%
61–75	13,701 11.7%	65,359 55.6%	38,507 32.8%	117,567 100%
76–90	2225 4.5%	18,987 38.5%	28,070 57%	49,282 100%
90 plus	201 3.8%	1637 30.5%	3521 65.7%	5359 100%
Sex; missing values *n* = 0				
Male	169,052 44.1%	168,677 44%	45,729 11.9%	45,729 11.9%
Female	169,670 38.5%	207,216 47%	64,030 14.5%	440,916 100%
Scottish Index of Multiple Deprivation; missing values *n* = 19,082 (2.3%)				
1	30,755 35.7%	41,730 48.5%	13,571 15.8%	86,056 100%
2	26,708 36.1%	35,288 47.7%	11,924 16.1%	73,920 100%
3	28,575 37.4%	35,732 46.8%	12,050 15.8%	76,357 100%
4	28,106 38.3%	34,003 46.3%	11,257 15.3%	73,366 100%
5	30,064 39.7%	34,634 45.8%	11,002 14.5%	75,700 100%
6	34,264 42.3%	36,724 45.3%	10,075 12.4%	81,063 100%
7	36,838 41.3%	40,844 45.7%	11,601 13%	89,283 100%
8	33,476 43.7%	34,293 44.7%	8882 11.6%	76,651 100%
9	32,405 43%	34,089 45.2%	8854 11.8%	75,348 100%
10	47,717 48.9%	40,745 41.8%	9086 9.3%	97,548 100%

### LTCs and risk of missing appointments

**Fig. 1:**
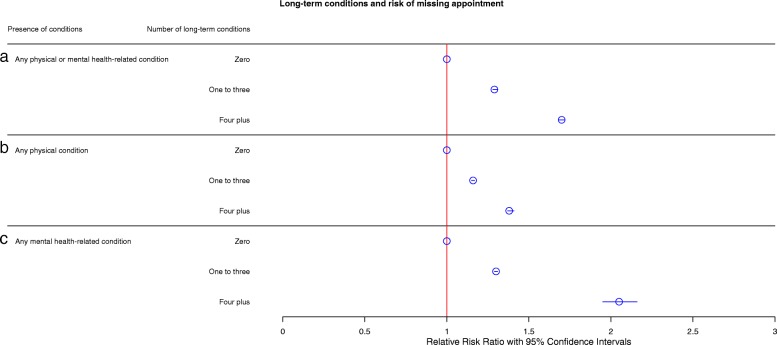
**a** Fully adjusted negative binomial modelling of risk of missing appointments for no, one to two, and four plus long-term conditions. Model controlled for age, sex, socioeconomic status (SIMD), distance between home and the practice, appointment delay, mean appointment time per patient, number of appointments per patient, rurality index, and mean practice socioeconomic status. The model is also offset for the number of appointments made. Circles represent relative risk ratios (RRRs) with 95% confidence intervals. **b** Fully adjusted negative binomial modelling of risk of missing appointment for physical health-related long-term conditions. Model controlled for age, sex, socioeconomic status, distance between home and the practice, appointment delay, mean appointment time per patient, number of appointments per patient, rurality index, mean practice socioeconomic status, and number of mental health-related long-term conditions. **c** Fully adjusted negative binomial modelling of risk of missing appointment for mental health-related long-term conditions. Model controlled for age, sex, socioeconomic status, distance between home and the practice, appointment delay, mean appointment time per patient, number of appointments per patient, rurality index, mean practice socioeconomic status and number of physical long-term conditions. All models are also offset for the number of appointments made. Circles represent RRRs with 95% confidence intervals

We next examined the relationship between risk of missing appointments and number of LTCs. Relative risks of missing appointments based on LTC groups are shown in Fig. 1a. Compared with the reference group of patients with LTC scores of zero (relative risk ratio (RRR) 1.00) in this model, patients with one to three LTCs were approximately 30% more at risk of missing appointments (RRR 1.29, 95% CI 1.28–1.31) and patients with four or more LTCs were 70% more at risk of missing appointments (RRR 1.70, 95% CI 1.68–1.72). All models are proportioned to the total number of appointments made per patient, ensuring that any increase in the total number of appointments made does not account for an increased risk of missing appointments.

To examine whether patients who have multiple appointments in a short timeframe are likely to miss appointments, we studied whether patients who missed an appointment had another within 30 days of that appointment (Additional file 2). Surprisingly, patients in the ‘high’ group had a lower number of appointments within 30 days of a missed appointment (5.7%) than the ‘low’ group (21.6%).

### Physical and mental health-reated LTCs

To provide further information on the type of LTC that increases the risk of missing appointments, we categorised LTCs into physical or mental health-related LTCs and examined the RRR for missed appointments for these types of LTCs alone. Physical health LTCs (Fig. 1b) were associated with a modest increased risk of missing appointments, with patients with one to three physical morbidities being 16% more likely to miss appointments (RRR 1.16, 95% CI 1.15–1.17) and those with four or more being approximately 38% more at risk of missing appointments (RRR 1.38, 95% CI 1.37–1.41). Mental health-related LTCs (Fig. 1c) represented a greater risk of missed appointments, with those with one to three mental morbidities being 30% more at risk of missing appointments (RRR 1.30, 95% CI 1.29–31), and those with four or more mental LTCs representing a greater than 2-fold adjusted increase in likelihood of missed appointments (RRR 2.05, 95% CI 1.95–2.16).

Next, we examined subtypes of mental health-related LTCs and their contribution towards missed appointments (Additional file 3). Both problem alcohol (22.2%) and psychoactive substance misuse (27.4%) contained a high proportion of patients who missed two or more appointments per year. Other mental health-related LTCs (depression, anxiety, dementia, schizophrenia and anorexia bulimia) appeared to have a smaller proportion of patients in the ‘high’ group, with only 14.1% missing two or more appointments per year. Patients with both alcohol misuse and other mental health-related LTCs showed a modest increase in proportion of patients in the ‘high’ group (25.4%), with a similar increase reported for those with both psychoactive substance misuse and other mental health-related LTCs (30.0%). The proportion of patients in the ‘high’ group appeared to be greatest in patients who suffered from both problem alcohol and psychoactive substance misuse (32.1%).

### All-cause mortality in missed appointments

**Fig. 2:**
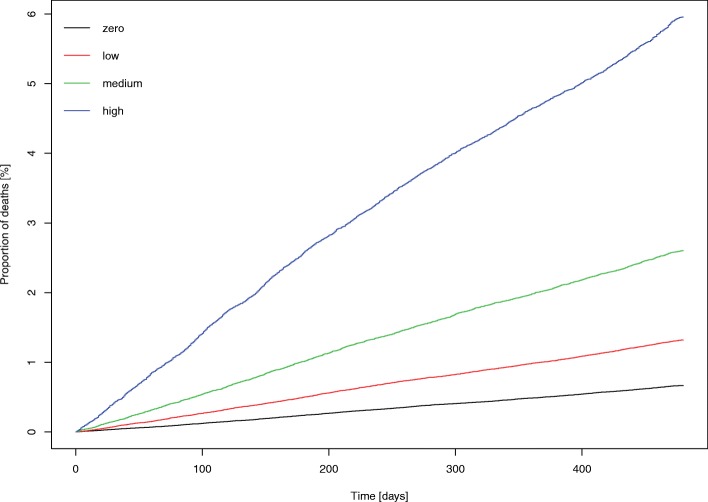
Cumulative incidence Kaplan–Meier plot showing proportions of deaths (all-cause mortality) over the follow-up period of 480 days. Graph shows zero, low, medium and high number of missed appointment groupings. Missed appointment categories were defined as the average annual number of missed appointments over a 3-year period, as follows: zero, 0; low, < 1; medium, 1–2; or high, > 2

**Fig. 3:**
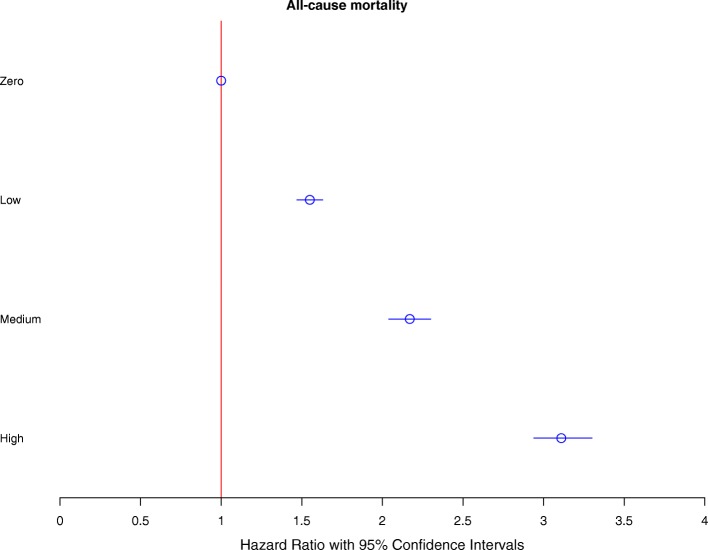
Fully adjusted Cox’s proportional hazards showing risk of all-cause mortality for zero, low, medium and high number of missed appointment groupings. Model controlled for age, sex, socioeconomic status (SIMD), distance between home and the practice, appointment delay, mean appointment time per patient, number of appointments per patient, rurality index, mean practice socioeconomic status and number of long-term conditions. Missed appointment categories were defined as the average annual number of missed appointments over a 3-year period, as follows: zero, 0; low, < 1; medium, 1–2; or high, > 2. Graph shows hazard ratios (HRs) with 95% confidence intervals

Using data linkage, we examined all-cause mortality in missed appointment groups. Figure 2 shows a cumulative incidence Kaplan–Meier plot containing proportions of deaths in the ‘zero’, ‘low’, ‘medium’ and ‘high’ groups, highlighting a clear trend toward an increased proportion of deaths in the ‘low’, ‘medium’ and ‘high’ groups compared with the ‘zero’ group. To further quantify the association, we performed Cox’s proportional hazards analysis to find the risk of all-cause mortality in each of the missed appointment groups using zero missed appointments as a reference (Fig. 3). This analysis controlled for number of LTCs, age, sex, socioeconomic status, distance to practice and practice-based factors (average appointment delay, average appointment duration, average appointments offered to each patient over 3 years, rurality and mean practice socioeconomic status). There was a dose-related increased risk of all mortality outcomes, with a greater than 50% increase in the ‘low’ group (hazard ratio (HR) 1.55, 95% CI 1.47–1.63), an over 2-fold increase in the ‘medium’ group (HR 2.17, 95% CI 2.04–2.30), and an approximately 3-fold increase in the ‘high’ group (HR 3.11, 95% CI 2.94–3.30).

**Fig. 4:**
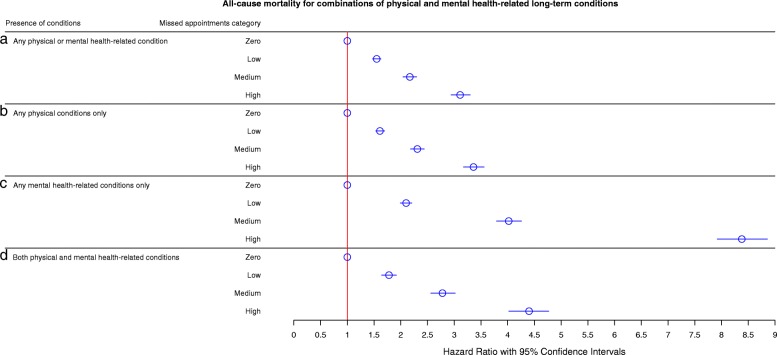
Fully adjusted Cox’s proportional hazards showing risk of all-cause mortality for zero, low, medium and high missed appointments groupings in (**a**) patients with any physical or mental health long-term conditions, (**b**) patients with any physical conditions only, (**c**) patients with mental health conditions only, and (**d**) patients with both physical and mental health conditions. Model controlled for age, sex, socioeconomic status (SIMD), distance between home and the practice, appointment delay, mean appointment time per patient, number of appointments per patient, rurality index, mean practice socioeconomic status, and number of long-term conditions. Missed appointment categories were defined as the average annual number of missed appointments over a 3-year period, as follows: zero, 0; low, < 1; medium, 1–2; or high, > 2. Graph shows hazard ratios (HRs) with 95% confidence intervals

To interpret the differing effects of physical and mental health on all-cause mortality, we examined all-cause mortality outcomes on groups of patients who had only physical health-related LTCs and those who had only mental health-related LTCs (Fig. 4a). This showed a dose-related effect for any physical or mental health-related LTC, with a stepwise increase in all-cause mortality for the ‘low’ (HR 1.55, 95% CI 1.47–1.63), ‘medium’ (HR 2.04, 95% CI 2.17–2.30) and ‘high’ (HR 3.11, 95% CI 2.91–3.30) groupings. Patients with physical health-related LTCs (Fig. 4b) showed similar patterns (low: HR 1.53, 95% CI 1.61–1.70; medium: HR 2.31, 95% CI 2.18–2.44; high: HR 3.36, 95% CI 3.17–3.56), likely due to the increased predominance of physical over mental health-related LTCs. The effects were more pronounced for mental health-related LTCs (Fig. 4c), with the ‘low’ group showing greater than double all-cause mortality (HR 2.10, 95% CI 1.99–2.21), the ‘medium’ group showing an over 4-fold increase (HR 4.02, 95% CI 3.79–4.25), and the ‘high’ group showing an over 8-fold increase (HR 8.37, 95% CI 7.91–8.87). This effect was less marked in patients with both mental and physical health-related LTCs (Fig. 4d), wherein the ‘low’ group showed an approximately 80% increased risk of all-cause mortality (HR 1.78, 95% CI 1.64–1.92), the ‘medium’ group showed an over 2.5-fold increase (HR 2.78, 95% CI 2.56–3.02) and the ‘high’ group showed an over 4-fold increase (HR 4.40, 95% CI 4.02–4.77).

### Causes and age at death in LTC groups

After examining the differential effect of physical or mental health-related LTCs and missed appointments on all-cause mortality, we next examined mean age at death and the most common primary causes of death in patients with no, mental only, physical only, or both mental and physical health-related LTCs (Table 2). The lowest mean age at death (49.3 years old) was in the ‘high’ group of patients with mental health-related LTCs alone. Conversely, patients in the ‘high’ group with physical health LTCs alone were, on average, approximately 80 years old at the time of death. Whilst patients experiencing either physical alone or physical and mental health-related LTCs lived to between 75 and 79 years old on average, those who died in the follow-up period with mental but no physical health-related LTCs lived to between 49 and 68 years old on average. The most common primary causes of death differed between groups, wherein those for patients with physical health-related LTCs alone were lung malignancy and ischaemic heart disease, and patients with both physical and mental health-related LTCs most commonly died with dementia or ischemic heart disease. In contrast, patients with mental health-related LTCs alone frequently had primary causes of death recorded as intentional self-harm by hanging, strangulation and suffocation, poisoning/overdoses, or an ill-defined or unknown cause of mortality (R99). Notably, patients with mental health-related LTCs alone are the only group that had the primary cause of death listed within the ‘external causes’ classification of ICD-10 codes in a very substantial proportion of cases [13].

**Table 2:** Number of deaths, mean age at death and common primary causes of death for groups with no long-term conditions, only mental health-related long-term conditions, only physical long-term conditions, and both physical and mental health-related long-term conditions

	No long-term conditions		
Missed appointment category	Number of deaths (% of group dead)	Mean age at death (SD)	Most common primary causes of death (%)
Zero	262 (0.1%)	68.06 (21.09)	I219 (8.4), C349 (5.7), R99 (5.7)
Low	119 (0.1%)	64.38 (21.78)	R99 (10), G309 (9.2), I259 (5)
Medium	41 (0.2%)	62.56 (23.08)	C349 (9.8), R99 (9.8), C221 (7.3)
High	24 (0.4%)	56.79 (27.14)	R99 (25), F019 (8.3), N40 (8.3)
	Only mental health-related long-term conditions		
Missed appointment category	Number of deaths (% of group dead)	Mean age at death (SD)	Most common primary causes of death
Zero	69 (0.2%)	55.72 (20)	R99 (11.6), X70 (10.1), I219 (8.7)
Low	83 (0.4%)	54.68 (18.79)	R99 (21.6), X70 (12), I219 (6)
Medium	58 (0.6%)	53.1 (20.18)	R99 (19), X42 (6.9), Y14 (6.9)
High	53 (1.7%)	49.3 (20)	R99 (32), G309 (9.4), Y14 (5.6)
	Only physical long-term conditions		
Missed appointment category	Number of deaths (% of group dead)	Mean age at death (SD)	Most common primary causes of death
Zero	1399 (0.1%)	77.12 (12.34)	C349 (8.3), I219 (7.3), I259 (3.2)
Low	1361 (1.9%)	77.46 (13.36)	I219 (7.3), C349 (6), I259 (4.2)
Medium	1025 (3.2%)	78.93 (12.54)	C349 (8.1), I219 (6.4), I259 (4.6)
High	1241 (6.6%)	79.97 (13.27)	C349 (6.1), I219 (5.8), I259 (4.2)
	Both physical and mental health-related long-term conditions		
Missed appointment category	Number of deaths (% of group dead)	Mean age at death (SD)	Most common primary causes of death
Zero	1193 (2.0%)	76.65 (13.53)	G309 (7.5), F03 (6.9), I219 (6.2)
Low	1432 (2.9%)	76.56 (13.59)	G309 (6.7), F03 (6.2), I219 (5.5)
Medium	1372 (4.2%)	75.01 (14.93)	G309 (6), F019 (5.8), I219 (5.3)
High	2114 (7.0%)	76.19 (15.29)	F019 (7.9), G309 (6.4), F03 (5.9)

Key: *I219* Acute myocardial infarction, unspecified; *C349* Malignant neoplasm of unspecified part of bronchus or lung; *R99* Ill-defined and unknown cause of mortality; *G309* Alzheimer’s disease, unspecified; *I259* Chronic ischemic heart disease, unspecified; *C221* Intrahepatic bile duct carcinoma; *F019* Vascular dementia, unspecified; *N40* Benign prostatic hyperplasia; *X70* Intentional self-harm by hanging, strangulation and suffocation; *X42* Accidental poisoning by and exposure to narcotics and psychodysleptics [hallucinogens], not elsewhere classified; *Y14* Poisoning by and exposure to other and unspecified drugs, medicaments and biological substances, undetermined intent; *F03* Unspecified dementia, *SD* standard deviation

## Discussion

We have described a strong association between the burden of multimorbidity and the likelihood of missing primary care appointments in a large, broadly representative sample of Scottish general practices. In the group of patients missing two or more appointments per year, almost half (46%) had one or more LTCs, while 17% had four or more. Furthermore, patients with four or more LTCs were more than twice as likely to miss appointments as those with none, even after controlling for multiple patient and practice factors. Those least likely to attend were patients with mental health-related LTCs.

Repeated missed appointments were also associated with substantially increased premature all-cause mortality rates, particularly amongst those with mental health-related LTCs. Among patients with physical health-related LTCs alone, those missing two or more appointments per year had a 3-fold increase in all-cause mortality compared to those who missed no appointments. For those with only mental health-related LTCs, the corresponding increase was more than 8-fold. A significant proportion of patients with alcohol or substance misuse appeared to serially miss appointments, with approximately one-quarter of each failing to attend two or more appointments per year, and approximately one-third of those with both failing to attend two or more appointments per year.

Unsurprisingly, among those with no recorded physical health LTCs, deaths occurred, on average, at younger ages than among those with physical health LTCs, but the age at death was lower with each escalating category of frequency of missed appointments; deceased patients with only mental health-related LTCs who missed two or more appointments per year died on average at age 49.

There are some limitations to this study. We were unable to establish the cause of each missed appointment directly. Some missed appointments, for example, may have resulted from patients being too sick to leave home. Nevertheless, missing multiple appointments still provides a risk marker of all-cause mortality even after controlling for a variety of other factors. While some conditions, such as dementia or Alzheimer’s disease, could also cause patients to forget appointments, these specific diagnoses only accounted for a small number of patients in our sample (*n* = 8927, 3.7%). In comparison, approximately 70% of patients with any combination of mental health multimorbidities suffered from depression, and almost half (49.2%) suffered from anxiety. Finally, there may be a proportion of patients who left Scotland and/or died during the follow-up period, which we are unable to account for.

Despite these limitations, we have established that a pattern of repeatedly missing appointments is a potentially valuable clinical marker for increased risk of premature mortality, particularly among those with mental health problems. It is unlikely that the relationship between missed appointments and mortality is directly causal in most cases, particularly among those without pre-existing physical health LTCs. One possible mechanism involves conditions associated with cognitive difficulties such as dementia, attention-deficit/hyperactivity disorder, or problem drug or alcohol use, each of which is associated with an increased risk of missing appointments and with increased mortality [14–20]. The latter two conditions are likely to underlie a substantial proportion of the premature deaths from external causes among patients with mental health-related LTCs who missed appointments frequently. Nevertheless, it remains possible that missed appointments among those with physical LTCs do contribute to the observed increased mortality [9].

## Conclusions

The main clinical implication of these findings is that general practices, the acute sector and other NHS services that support patients should consider how best to facilitate engagement of patients with patterns of repeatedly missing appointments. Our previous work suggested an association between rates of non-attendance and an increasing time gap from appointment booking to appointment date [10]. Therefore, there is a case for conducting exploratory studies similar to small-scale research interventions performed in mental health services involving a system of on-the-day appointments [21] for selected patients.

## Additional files

Additional file 1: **Table S1.** Categories of variables used in this paper and descriptions of the content of each variable. (DOCX 14 kb)

Additional file 2: **Table S2.** Secondary appointment status within 30 days of first appointment by patient attendance category. (DOCX 14 kb)

Additional file 3: **Table S3.** Missed appointment categories for patients with problem alcohol use; with problem psychoactive substance misuse; with other mental health conditions; with both problem alcohol and substance misuse; with both problem alcohol misuse and other mental health conditions; and with both problem psychoactive substance misuse and other mental health conditions. (DOCX 16 kb)
