# Supplementary material for: Morbidity, mortality and missed appointments in healthcare: a national retrospective data linkage study
Source: BMC Med. 2019 Jan 11;17:2. doi: 10.1186/s12916-018-1234-0 (PMC6329132; doi:10.1186/s12916-018-1234-0)
Supplement: Supplementary file 1 — Table S1. Categories of variables used in this paper and descriptions of the content of each variable. (DOCX 14 kb) [file 12916_2018_1234_MOESM1_ESM.docx]

Table S1 - Categories of data available

| **Variable** | **Description** |
| --- | --- |
| Patient ID | Randomised ID of patient |
| Practice ID | Randomised ID of practice |
| Read code | Purpose of patients appointment |
| Attended | Did patient attend appointment? |
| Category of attendance | Zero, low, medium or high attendance category |
| Age | Age of patient at time of extract |
| Sex | Sex of patient |
| Registration status | Registration status of patient at practice |
| Hospital distance | Distance between patient’s home and hospital |
| Practice distance | Distance between patient’s home and practice |
| Rural 8 | Urban/Rural categorisation of patient’s home |
| Practice rural 8 | Urban/Rural categorisation of patient’s practice |
| SIMD | Scottish index of multiple deprivation score for patient’s home |
| Practice SIMD | Scottish index of multiple deprivation score for patient’s practice |
| Pain prescriptions | Pain prescriptions taken by patient |
| Asthma prescriptions | Asthma prescriptions taken by patient |
| Anxiety prescriptions | Anxiety prescriptions taken by patient |
| IBS prescriptions | IBS prescriptions taken by patient |
| Constipation prescriptions | Constipation prescriptions taken by patient |
| Migraine prescriptions | Migraine prescriptions taken by patient |
| NSAID prescriptions | Non-steroidal anti-inflammatory drug prescriptions taken by patient |
| Dyspepsia prescriptions | Dyspepsia prescriptions taken by patient |
| Lithium prescriptions | Lithium prescriptions taken by patient |
| Death | Patient death status |
| Follow up length | Duration of time of follow up for patient |
| Hypertension | Patient has or does not have this multimorbidity |
| Depression | Patient has or does not have this multimorbidity |
| Asthma | Patient has or does not have this multimorbidity |
| Coronary heart disease | Patient has or does not have this multimorbidity |
| Diabetes | Patient has or does not have this multimorbidity |
| Thyroid disorder | Patient has or does not have this multimorbidity |
| Rheumatoid arthritis | Patient has or does not have this multimorbidity |
| Hearing loss | Patient has or does not have this multimorbidity |
| Chronic obstructive pulmonary disease | Patient has or does not have this multimorbidity |
| Anxiety | Patient has or does not have this multimorbidity |
| Irritable bowel syndrome | Patient has or does not have this multimorbidity |
| Cancer | Patient has or does not have this multimorbidity |
| Alcohol problems | Patient has or does not have this multimorbidity |
| Psychoactive substance misuse | Patient has or does not have this multimorbidity |
| Stroke/transient ischemic attack | Patient has or does not have this multimorbidity |
| Chronic kidney disease | Patient has or does not have this multimorbidity |
| Atrial fibrillation | Patient has or does not have this multimorbidity |
| Peripheral vascular disease | Patient has or does not have this multimorbidity |
| Heart failure | Patient has or does not have this multimorbidity |
| Prostate disease | Patient has or does not have this multimorbidity |
| Glaucoma | Patient has or does not have this multimorbidity |
| Epilepsy | Patient has or does not have this multimorbidity |
| Dementia | Patient has or does not have this multimorbidity |
| Schizophrenia | Patient has or does not have this multimorbidity |
| Psoriasis/Eczema | Patient has or does not have this multimorbidity |
| Inflammatory bowel disease | Patient has or does not have this multimorbidity |
| Migraine | Patient has or does not have this multimorbidity |
| Blindness | Patient has or does not have this multimorbidity |
| Sinusitis | Patient has or does not have this multimorbidity |
| Learning disability | Patient has or does not have this multimorbidity |
| Anorexia/bulimia | Patient has or does not have this multimorbidity |
| Chronic bronchitis | Patient has or does not have this multimorbidity |
| Parkinson’s disease | Patient has or does not have this multimorbidity |
| Multiple sclerosis | Patient has or does not have this multimorbidity |
| Viral hepatitis | Patient has or does not have this multimorbidity |
| Chronic liver disease | Patient has or does not have this multimorbidity |
| Multimorbidity count | Count of number of multimorbidities |
| Physical MM count | Count of number physical of multimorbidities |
| Mental MM count | Count of number of mental multimorbidities |
