# Supplementary material for: Morbidity, mortality and missed appointments in healthcare: a national retrospective data linkage study
Source: BMC Med. 2019 Jan 11;17:2. doi: 10.1186/s12916-018-1234-0 (PMC6329132; doi:10.1186/s12916-018-1234-0)
Supplement: Supplementary file 2 — Table S2. Secondary appointment status within 30 days of first appointment by patient attendance category. (DOCX 14 kb) [file 12916_2018_1234_MOESM2_ESM.docx]

Table S2

| *Attendance Category* | *Appointment within 30 days* | | ***Total*** |
| --- | --- | --- | --- |
|  | no | yes |  |
| low | 235423 78.4 % 16 % | 65027 21.6 % 39.5 % | 300450 100 % 18.4 % |
| medium | 343911 88.1 % 23.4 % | 46378 11.9 % 28.1 % | 390289 100 % 23.9 % |
| high | 889785 94.3 % 60.6 % | 53360 5.7 % 32.4 % | 943145 100 % 57.7 % |
| ***Total*** | 1469119 89.9 % 100 % | 164765 10.1 % 100 % | 1633884 100 % 100 % |
| *χ^2^=66046.208 · df=2 · Cramer's V=0.201 · p=0.000* | | | |
