# Supplementary material for: Morbidity, mortality and missed appointments in healthcare: a national retrospective data linkage study
Source: BMC Med. 2019 Jan 11;17:2. doi: 10.1186/s12916-018-1234-0 (PMC6329132; doi:10.1186/s12916-018-1234-0)
Supplement: Supplementary file 3 — Table S3. Missed appointment categories for patients with problem alcohol use; with problem psychoactive substance misuse; with other mental health conditions; with both problem alcohol and substance misuse; with both problem alcohol misuse and other mental health conditions; and with both problem psychoactive substance misuse and other mental health conditions. (DOCX 16 kb) [file 12916_2018_1234_MOESM3_ESM.docx]

|  | **Zero missed appointments (%)**  **N=439,592** | **Low missed appointments (%)**  **N=228,365** | **Medium missed appointments (%)**  **N=97,077** | **High missed appointments (%)**  **N=59,340** | **Total (%)**  **N=824,374** |
| --- | --- | --- | --- | --- | --- |
| **Problem alcohol misuse; missing values n= 0** | | | | | |
| **Yes** | 6196 (27.3 %) | 6297 (27.7 %) | 5171 (22.8 %) | 5053 (22.2 %) | 22717 (100 %) |
| **No** | 433396 (54.1 %) | 222068 (27.7 %) | 91906 (11.5 %) | 54287 (6.8 %) | 801657 (100 %) |
| **Problem psychoactive substance misuse; missing values n= 0** | | | | | |
| **Yes** | 4016 (21.3 %) | 4953 (26.2 %) | 4737 (25.1 %) | 5178 (27.4 %) | 18884 (100 %) |
| **No** | 435576 (54.1 %) | 223412 (27.7 %) | 92340 (11.5 %) | 54162 (6.7 %) | 805490 (100 %) |
| **Other mental health conditions; missing values n= 0** | | | | |  |
| **Yes** | 90984 (39.1 %) | 68729 (29.5 %) | 40377 (17.3 %) | 32856 (14.1 %) | 232946 (100 %) |
| **No** | 348608 (58.9 %) | 159636 (27 %) | 56700 (9.6 %) | 26484 (4.5 %) | 591428 (100 %) |
| **Both problem alcohol and substance misuse; missing values n=0** | | | | | |
| **Yes** | 732 (17.3 %) | 992 23.4 %) | 1152 (27.2 %) | 1359 (32.1 %) | 4235 (100 %) |
| **No** | 438860 (53.5 %) | 227373 (27.7 %) | 95925 (11.7 %) | 57981 (7.1 %) | 820139 (100 %) |
| **Both problem alcohol misuse and other mental health conditions; missing values n=0** | | | | | |
| **Yes** | 3796 (24 %) | 4231 (26.8 %) | 3767 (23.8 %) | 4007 (25.4 %) | 15801 100 %) |
| **No** | 435796 (53.9 %) | 224134 (27.7 %) | 93310 (11.5 %) | 55333 (6.8 %) | 808573 (100 %) |
| **Both problem psychoactive substance misuse and other mental health conditions; missing values n=0** | | | | | |
| **Yes** | 2606 (18.9 %) | 3515 (25.5 %) | 3549 (25.7 %) | 4138 (30 %) | 13808 (100 %) |
| **No** | 436986 (53.9 %) | 224850 (27.7 %) | 93528 (11.5 %) | 55202 (6.8 %) | 810566 (100 %) |
